# H1N1 Influenza Causing Fulminant Myocarditis Requiring Extracorporeal Membrane Oxygenation

**DOI:** 10.7759/cureus.4665

**Published:** 2019-05-14

**Authors:** Ali Hamoudi, Dana Vais, Vian Taqi

**Affiliations:** 1 Internal Medicine, Chicago Medical School / Rosalind Franklin University of Medicine and Science, Chicago, USA; 2 Infectious Disease, AMITA Saints Mary and Elizabeth Medical Center / Rosalind Franklin University of Medicine and Science, Chicago, USA; 3 Internal Medicine, University of Baghdad, Baghdad, IRQ

**Keywords:** h1n1, myocarditis, ecmo, vasopressors

## Abstract

Influenza infection is a known cause of global morbidity and mortality. Most cases of influenza A (H1N1) influenza infection are mild and do not require hospitalization. Although the most common presentation is with upper respiratory tract symptoms, hemodynamic instability requiring vasoactive drugs and ventilatory support use is unusual. We present a case of acute fulminant myocarditis that presented with dyspnea, which was confirmed with laboratory tests, chest X-ray, and echocardiogram. The test for H1N1 in nasopharyngeal secretions was positive. The patient evolved to refractory cardiogenic shock despite the clinical measures applied.

## Introduction

Acute myocarditis is defined as an inflammation of the myocardial muscle, causing myocytes necrosis and inflammatory infiltrate of the heart muscle. The most common etiology is attributed to viral infections like Coxsackie B virus and adenovirus.

Many people are affected by seasonal influenza every year, causing symptoms that are limited to the respiratory system. Myocardial involvement by seasonal influenza virus seems to be low [1-2].

The frequency of myocarditis secondary to seasonal influenza virus infection ranges from 0%-11%, depending on the criteria used to diagnose myocarditis, however, the prevalence of influenza A (H1N1) myocarditis remains unclear [3]. In addition to the direct effect of influenza virus infection, pro-inflammatory cytokines are thought to be responsible for the pathogenesis of cardiac dysfunction.

Due to the high affinity for cardiac myocytes, the Coxsackie B virus has been reported as the most common pathogen of viral myocarditis. The higher affinity of the virus with widespread myocardial involvement by the Coxsackie B virus is compared to the lower affinity of the influenza virus, which tends to be localized.

Most documented cases of H1N1 myocarditis were reported during the pandemic season 2009-2010 in Asia, with a significant drop in reported cases in the subsequent years.

The clinical severity of the H1N1 infection, ranging from asymptomatic to fulminant myocarditis, causes severe hemodynamic dysfunction, necessitating vasopressors use and mechanical circulatory support. The worst outcome may end up in death due to impaired cardiac function, although such fulminant myocarditis associated with influenza infection is rare [4-5]. Our patient had an unfortunate outcome following a recent H1N1 infection despite all supportive measures.

## Case presentation

We present a 25-year-old African American female with no relevant past medical history, who presented with shortness of breath and sharp central chest pain. The patient stated that she had flu-like symptoms, including fatigue, sore throat, headaches, low-grade fever, and generalized body aches that started one week before admission. Although she took over-the-counter (OTC) flu remedies (e.g. Theraflu) and multiple non-steroidal anti-inflammatory drugs (NSAIDs); her symptoms progressively worsened to the point that she wasn’t able to walk more than a few steps due to dyspnea and fatigue. The patient denied any recent travel, sick contact, or recent influenza immunization. She smoked one pack of tobacco per day and drank alcohol socially.

**Figure 1:**
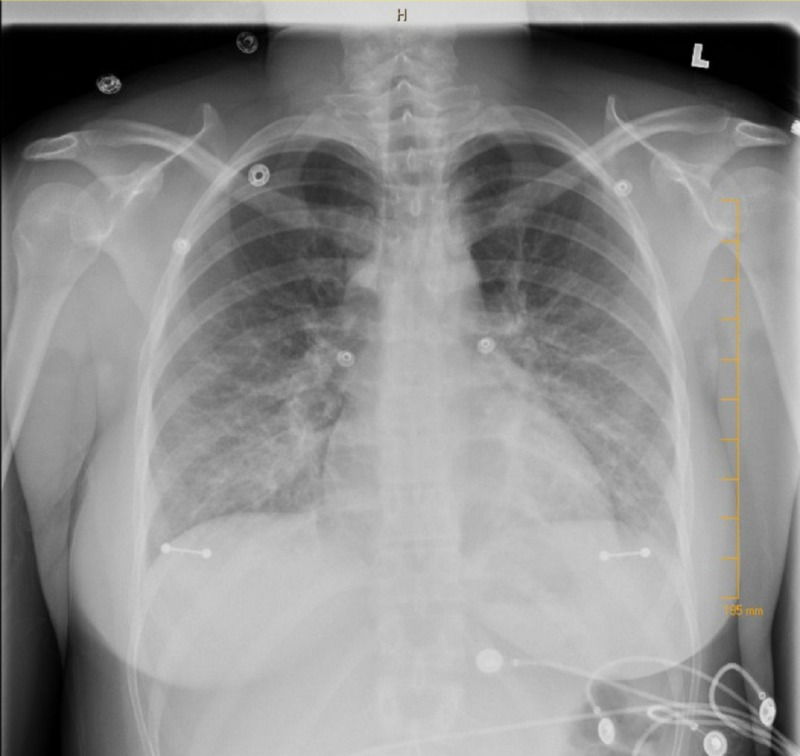
Pulmonary edema

In the emergency department (ED), her temperature was 98.6 °F (37 °C), heart rate 106, respiratory rate (RR) 18, and blood pressure (BP) 102/66. On physical exam, the patient was lethargic and tired without significant physical findings. Complete blood count (CBC) showed no leukocytosis, comprehensive metabolic panel (CMP) was normal except for mild hyperglycemia (155 mg/dl), and urine toxicology was negative. Antinuclear antibodies (ANA) was negative, thyroid stimulating hormone (TSH) was normal (0.54), and D-Dimer was normal. Serial electrocardiograms (EKGs) revealed sinus tachycardia with diffuse ST segment elevations and serial troponins steadily increased (2.51->2.94->3.55). Brain natriuretic peptides (BNP) was elevated (163 pg/mL) and creatinine kinase (CK) (501). The respiratory viral panel was positive for influenza A subtype H1-2009. Coxsackie A and B, parvovirus B19, immunoglobulin M (IgM), and human immunodeficiency virus (HIV) tests were negative. Chest X-ray revealed mild pulmonary edema (Figure 1). The patient was admitted to the intensive care unit (ICU) and started on oseltamivir, frusemide, and spironolactone.

During her hospital stay, her condition got worse and she felt progressively cold, sweaty, with increasing shortness of breath and blood pressure continuing to run on the low side. She developed bilateral lung crackles with bilateral leg pitting edema with chest X-ray (CXR) showed worsening pulmonary edema with bilateral pleural effusions. The arterial line showed worsening cardiac index from 2.4 to 1.3 and the patient was diagnosed with cardiogenic shock and started on vasopressors (dobutamine and norepinephrine). Diuretics were held. Transthoracic echo showed normal-sized left ventricle with diffuse hypokinesis and a left ventricular ejection fraction (LVEF) of 35% with type one diastolic dysfunction of the left ventricle. Despite using vasopressors, her condition did not improve. The patient then was transferred to a tertiary center where she had extracorporeal membrane oxygenation (ECMO) placed while waiting for a cardiac transplant. However, her condition continued to deteriorate and she passed away after that.

## Discussion

The presentation of acute myocarditis is usually variable and ranges from asymptomatic to acutely decompensated heart failure. The most common presenting symptom is dyspnea, which usually presents between Days 4 and 7 following the initial symptoms of viral infection [6]. Other cardiac symptoms, including chest pain, syncope, hypotension, and arrhythmia, have also been reported.

Fulminant myocarditis is one of the major causes of acute-onset heart failure and is responsible for approximately 10%-20% of cases of sudden death among young patients affected by H1N1 influenza [7]. Other cardiac complications, like myocardial ischemia arrhythmias, pericardial effusion, and cardiac tamponade, have been documented. The diagnosis of myocarditis can be made with a combination of symptoms, elevated cardiac enzymes, and echocardiographic findings.

Most of these patients exhibit electrocardiogram (ECG) abnormalities such as ST-segment elevation. As damage to myocardial cells ensues, cardiac biomarkers like troponin and CK-MB start to rise in the blood. Elevation of serum interleukin-10 in patients with severe acute myocarditis may predict the need for mechanical cardiopulmonary support [8].

Echocardiography is the mainstay in making the diagnosis of acute myocarditis. Classical findings include diffuse or focal left ventricular wall motion abnormalities, resulting in a reduction in the ejection fraction in most of the affected patients.

Although endomyocardial biopsy has been the gold standard for making the pathologic diagnosis, it is not required for the majority of cases due to the patchy involvement of myocardial muscle. Cardiac magnetic resonance imaging is emerging as the imaging modality of choice for determining the activity and severity of myocardial inflammation as well as being able to localize sites for endomyocardial biopsies [9].

Cardiovascular magnetic resonance imaging (CMR) was used as the diagnostic tool in several cases with pericardial/myocardial involvement during H1N1pdm2009 infection.

Although the majority of cases of acute myocarditis are mild and ended up with a spontaneous improvement, some cases may be fatal. Many cases not recognized during the acute episode may develop into dilated cardiomyopathy later and may require cardiac transplantation [10]. Therefore, early recognition and treatment are of significant importance, especially during influenza pandemics. Despite the widespread of influenza pandemics, only a few cases of fulminant myocarditis from the USA and Europe have been reported [11-15].

The treatment of fulminant myocarditis is based on hemodynamic and ventilatory support. The use of antiviral agents has been shown to decrease morbidity and mortality in H1N1 viral infection. However, as this was a case of fulminant evolution, with rapid progression, antiviral treatment probably would not have had sufficient time to be effective. Hemodynamic support can be achieved through the use of various vasopressors to support circulation. Lung support with extracorporeal membrane oxygenation (ECMO) in addition to mechanical circulatory support (LVAD, intra-aortic balloon pump (IABP)) has been documented in cases not responding to initial vasopressors. Mechanical circulatory devices can be used to decrease mortality and as a bridge to transplantation. The use of immunosuppressive therapy in acute myocarditis of viral etiology is controversial.

## Conclusions

Physicians should stay alert and hyper-vigilant in identifying and treating influenza A (H1N1) and its complications as early as possible despite the H1N1 pandemic being over. Although the most common reason for hospital admission during the pandemic attack of H1N1 influenza was due to pneumonia, acute fulminant myocarditis due to sporadic influenza infection can still be identified.

Our case of fulminant myocarditis affecting African American young patients without a history of travel outside the United States happened after the pandemic season ended up in the unfortunate outcome of cardiogenic shock and death despite ECMO use. As a bottom line, physicians need to increase awareness of this rare and potentially fatal complication of influenza virus infection. Early identification with bedside ultrasound is crucial for early detection and therapeutic guidance, as the early employment of hemodynamic support can affect the final outcome.
